# Evaluation of the effect of three innovative recyling methods on the shear bond strength of stainless steel brackets-an in vitro study

**DOI:** 10.4317/jced.53586

**Published:** 2017-04-01

**Authors:** Neeraj Gupta, Dilip Kumar, Aparna Palla

**Affiliations:** 1 Assistant Professor, College of dental Sciences, Davangere, Karnataka, India; 2 Associate Professor, College of dental Sciences, Davangere, Karnataka, India

## Abstract

**Background:**

Orthodontists are commonly faced with the decision of what to do with debonded or inaccurately positioned brackets. An economical option to this dilemma is to recycle the brackets. Many recycling methods have been proposed, but the optimal bond strength of these recycled brackets needs further evaluation. Objectives: To evaluate and compare the effect of three recycling methods: (i) Sandblasting (ii) Sandblasting / direct flaming (iii) Sandblasting /direct flaming /acid bath solution on shear bond strength (SBS) of stainless steel brackets.

**Material and Methods:**

Eighty human premolars were bonded with premolar stainless steel brackets as per manufacturer’s instructions. The teeth were divided into 4 groups (n=20): Recycling and initial debonding was not done in Control group (Group I). After initial bonding, the brackets in the rest of the three experimental groups were debonded and recycled by following methods: (i) Sandblasting (Group II) (ii) Sandblasting /direct flaming (Group III) (iii) Sandblasting /direct flaming /acid bath solution (Group IV). Further the recycled brackets were bonded. The specimens were then subjected to testing in a Universal machine. The evaluation of the variation of the shear bond strength (SBS) among test groups was done using one-way ANOVA test and inter-experimental group comparison was done by Newman-Keuls multiple post hoc procedure.

**Results:**

Group I (8.6510±1.3943MPa) showed the highest bond strength followed by Group II (5.0185±0.9758MPa), Group IV (2.30±0.65MPa) and Group III (2.0455± 0.6196MPa). Statistically significant variations existed in the shear bond strength (SBS) in all groups analyzed except between Group III and Group IV.

**Conclusions:**

The following conclusions were drawn from the study: 1. Shear bond strength of new brackets is significantly higher than the recycled brackets. 2. Brackets sandblasted with 90µm aluminium oxide particle air-abrasion showed significantly higher shear bond strength compared to direct flaming/sandblasting and direct flaming/sandblasting/acid bath solution. 3. Sandblasting with 90µm aluminium oxide particle air-abrasion is the simplest, most efficient and hence, the preferred method of recycling debonded brackets.

** Key words:**Orthodontic bracket, recycling, shear bond strength.

## Introduction

In Orthodontics, as well as in other dental fields, there is a trend to simplify the technical procedures to reduce operative time and treatment costs. Before the 1970’s, orthodontic treatment with fixed appliances was performed using stainless steel bands that were cemented to all teeth and then orthodontics brackets were welded to the bands. The technique of bonding orthodontic accessories directly to tooth surfaces has become possible after Buonocore’s pioneer study (1). This resulted in the existence of a significantly stronger mechanical bond between restorative materials and dental enamel etched with 85% phosphoric acid for 30 seconds. The technique involving application of adhesive systems to acid-etched enamel allowed an optimal bonding of orthodontic brackets to tooth surface, which greatly improved and simplified the placement of fixed orthodontic appliances and widen the scopes and perspectives in Orthodontics (2).

The failure of a bonded orthodontic bracket during the course of therapy is not an uncommon occurrence (3). This is usually the consequence of either patients accidentally applying inappropriate force to the bracket or a poor bonding technique. Orthodontists are commonly faced with the decision of what to do with debonded or inaccurately positioned brackets that require repositioning during treatment (4). Thus, a significant number of teeth must be rebonded in a busy orthodontic practice. One solution is to recycle the brackets. The recycling process basically consists of removing bonding agent remnants from the bracket base, thus allowing the brackets to be reused without causing damage to the retention mesh and preserving its retentive characteristics.

## Material and Methods

-Objectives

1. To evaluate the effect of following three in-office recycling methods on shear bond strength of orthodontic brackets:

• Brackets sandblasted with aluminium oxide (90µm) abrasion.

• Brackets direct flamed followed by sandblasting.

• Brackets direct flamed,sandblasted followed by cleaning with acid bath solution (32% hydrochloric acid and 55% nitric acid, mixed in a 1:4 ratio) 

2. To compare the shear bond strengths of orthodontic brackets recycled by three different methods.

-Methodology

This *in vitro* study was carried out at the Department of Orthodontics. Eighty healthy human premolars extracted for orthodontic reasons were collected from Department of Pedodontics and Oral Surgery.

The following criteria were considered:

Inclusion criteria:

1. Intact buccal enamel.

Exclusion criteria: 

1. Premolars having developmental defects.

2. Cracks caused by the extraction forceps and caries.

•Research Design

This is an experimental *in vitro* study.

•Method of study:

Eighty premolar teeth which were extracted for orthodontic purpose were selected for this study. The teeth did not undergo pre treatment with a chemical agent such as alcohol, formalin or hydrogen peroxide. These teeth were thoroughly cleaned of any soft tissue and blood and stored immediately in saline to prevent dehydration till the study was conducted. Pre-adjusted edgewise premolar brackets of 0.022” (3M Unitek, Gemini M.B.T, Monorovia ,USA) were used in the study.

The teeth were divided into four groups:

Group I: New Brackets (Control group) in which no recycling was carried out.

Group II: Brackets recycled by sandblasting with aluminium oxide (90µm) abrasion.

Group III: Brackets recycled by direct flaming and sandblasting

Group IV: Brackets recycled by direct flaming, sandblasting followed by cleaning with acid bath solution (32% hydrochloric acid and 55% nitric acid, mixed in a 1:4 ratio).

Teeth in each group were mounted vertically on dental plaster blocks. The dental plaster bases were covered up to the usual level of alveolar bone around each premolar tooth. Teeth were aligned with the facial surface of the tooth perpendicular with the bottom of the mold; i.e., each tooth was oriented so its labial surface would be parallel to the force during the shear strength test. The teeth were kept outside the saline water only for a very short time to prevent any dehydration. The teeth were cleaned and then polished with non-fluoridated pumice and bristle brush for 15 seconds and air stream for 10 seconds.

ºBonding protocol

The bonding approach followed the manufacturer’s instructions. All eighty premolar teeth were bonded [Transbond XT(3M Unitek)]. The procedure included acid etching with a 37% phosphoric acid gel (EAZETECH, Anabond, Tamilnadu ) for 60 seconds followed by thorough washing and air drying for 20 seconds. The sealant was placed on the tooth, and the brackets [pre-adjusted edgewise premolar brackets of 0.022” (3M, M.B.T prescription)] were bonded with the adhesive and light cured (Unicorn MedidentPvt. Ltd, New Delhi) for 20 seconds. Before light curing the adhesive, the brackets were pressed on the tooth with and excess adhesive was removed with a sharp scaler.

ºDebonding procedure

Debonding was done with debonding pliers for the three experimental groups [Group II, Group III, Group IV]. In Group I (control), the bonded brackets remained attached to tooth surface until shear testing i.e. no debonding/rebonding procedures was done.

ºRecycling

Three different recycling methods following bracket debonding, were applied on the experimental groups to remove the resin layer to the bracket base prior to rebonding.

1. Group I:Control Group

2. Group II: Sandblasting (Santer Lobo 16, Confident Pvt. Ltd, Bangalore, Karnataka, India) with aluminium oxide (90µm) abrasion for 15-30 seconds (depending upon the residual bonding agent) with 10 mm distance from the tip and bracket base.

3. Group III: Direct flaming (600-800°C) till the adhesive became cherry red and then quenched in cold water followed by sandblasting.

4. Group IV: Direct flaming, sandblasting followed by cleaning with acid bath solution (32% hydrochloric acid and 55% nitric acid, mixed in a 1:4 ratio) for 5-15 seconds and thoroughly rinsed in running water between 30-60 seconds.

ºRebonding

The adhesive remaining on the teeth after debonding was removed with a tungsten carbide bur. Rebonding of the recycled bra-kets was done using standard bonding procedure as described earlier.

ºFinal debonding

A customized jig was suspended from the crosshead of a UNIVERSAL TESTING MACHINE (TUE-C-400, Fine Spavy Associates & Engineers Pvt. Ltd., Miraj). A gingivo-occlusal load was applied to the bracket, producing shear force at the bracket-tooth interface for all the four groups. A computer, electronically connected with the test machine, recorded the results of each test. Shear bond strengths were measured at a crosshead speed of 0.5 mm/ min.

**Figure 1:**
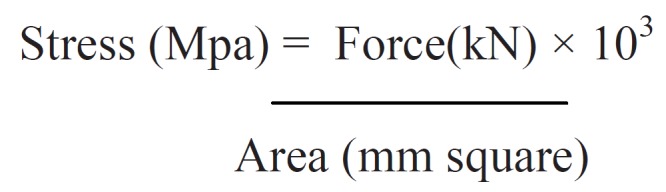
Equation.

The force required to break the bracket-enamel bond was recorded in Kilo Newtons (kN) and converted to megapascals (MPa) using the surface area of the bracket base. The following equation was used for the conversion, (Fig. 1).

Surface area of the bracket base was 9.8 mm2

ºData analysis

The data analysis was done using a statistical software SPSS 9.0.The shear bond strength values of the control and the experimental group was represented as Mean± Standard deviation.

The comparison of the shear bond strengths of the test groups was evaluated statistically using one way ANOVA. Pairwise comparison between the experimental groups was done by Newman-Keuls post hoc analysis. A probability value (*p* value) ≤ 0.05 was considered to be statistically significant.

## Results

 Table 1 shows the mean and standard deviation of the shear bond strength (SBS) values in the control group and the recycled groups. The values were 8.6510±1.3943MPa, 5.0185±0.9758MPa, 2.0455±0.6196MPa, .3020±0.65MPa for Group I, Group II, Group III, Group IV respectively.

**Table 1:**
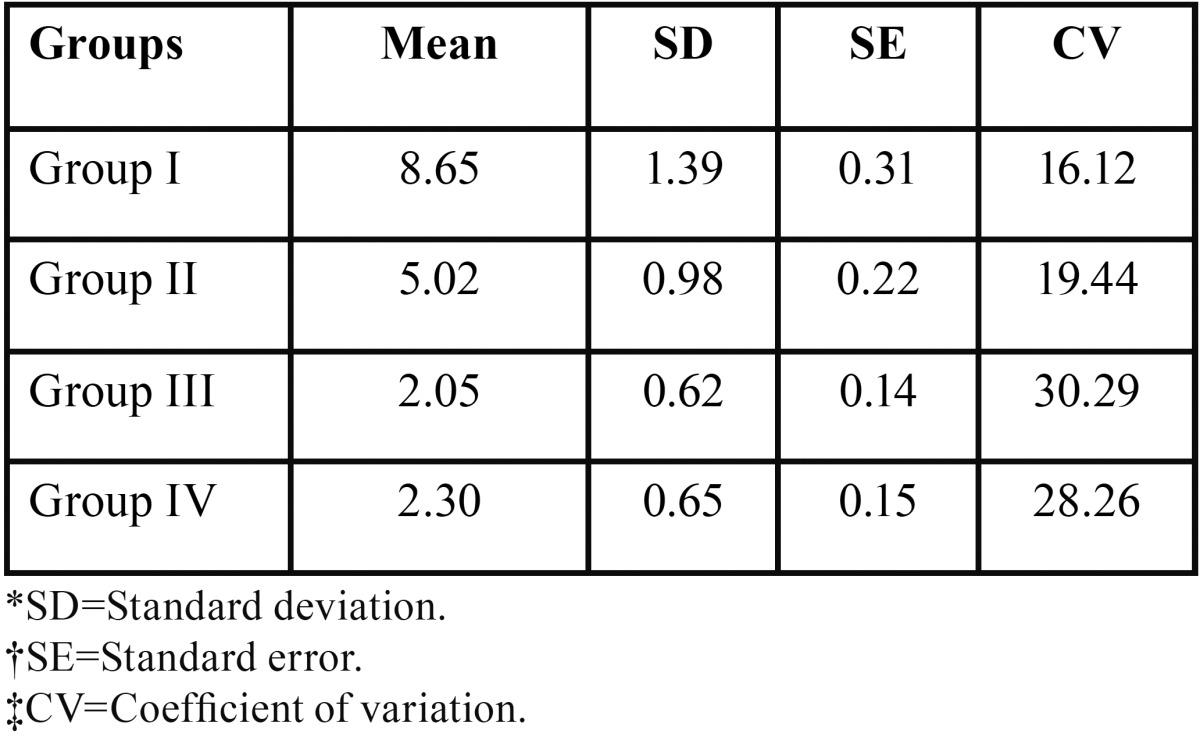
Mean Standard deviation, Standard error and Coefficient of variation of Shear bond strengths (MPa) in the test groups.

 Table 2 shows that the four test groups show a significant difference in the sher bond strengths (F=204.17) and *p* value of 0.00001. The difference between the four groups is highly significant statistically.

**Table 2:**
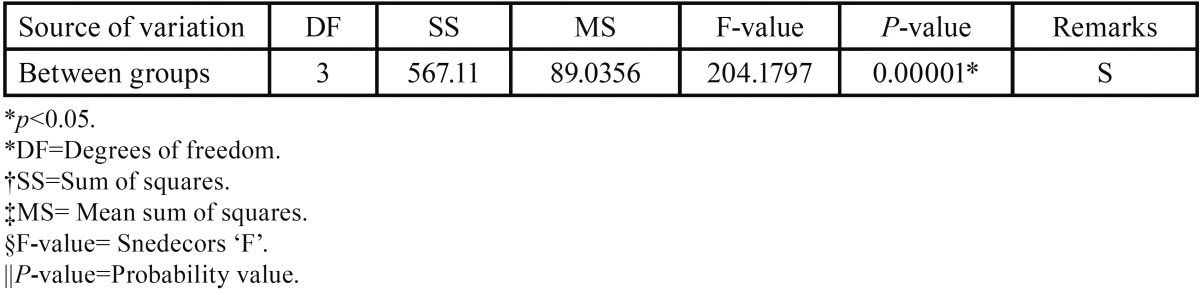
Statistical comparison of the shear bond strength of the test groups by one-way ANOVA.

**Figure 2:**
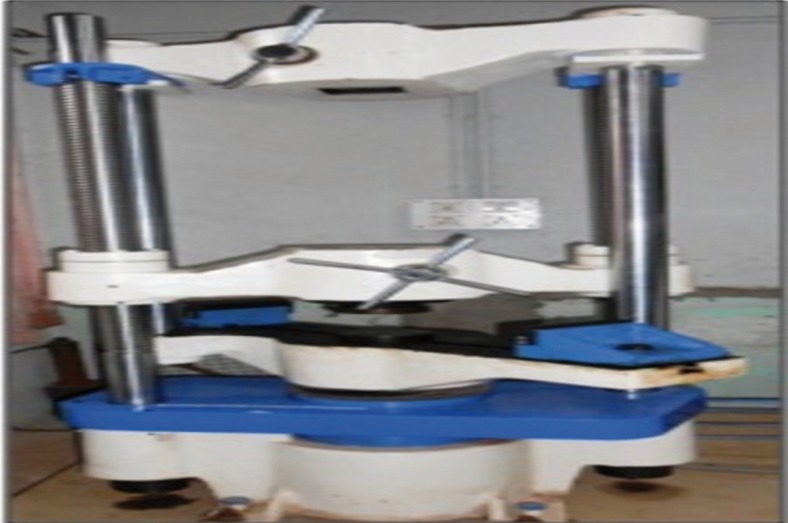
Universal testing machine setup.

**Figure 3:**
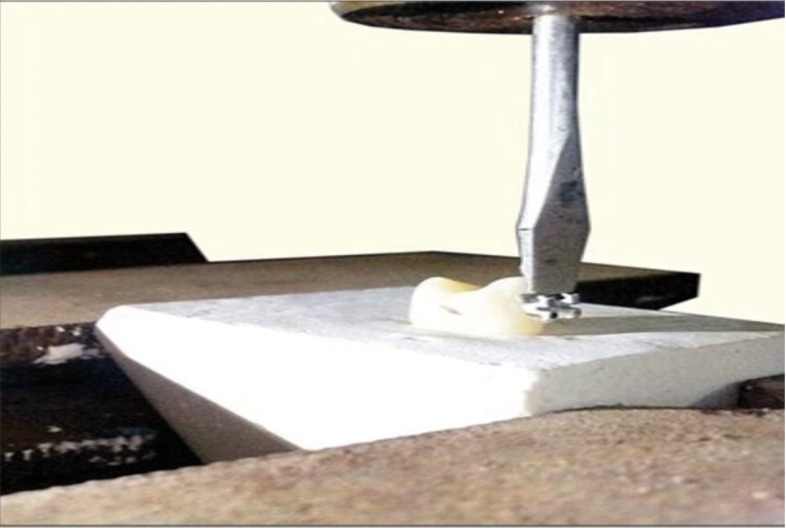
Customized jig producing shear force at the bracket-tooth interface.

**Figure 4:**
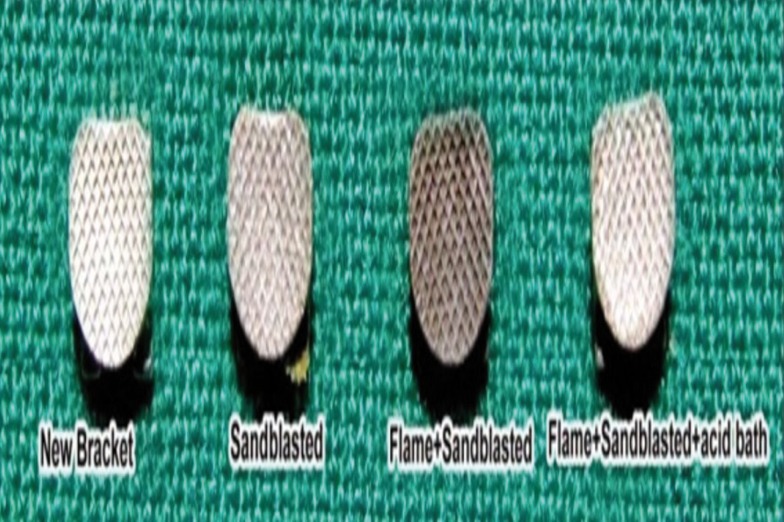
Appearance of the new and recycled bracket mesh bases.

 Table 3 shows the intergroup comparison between the four test groups. There was significant difference in the shear bond strength between the groups at 5% level of significance except between Group III and Group IV where the result was statistically non significant (*p*=0.4020), (Figs. 2-4).

**Table 3:**
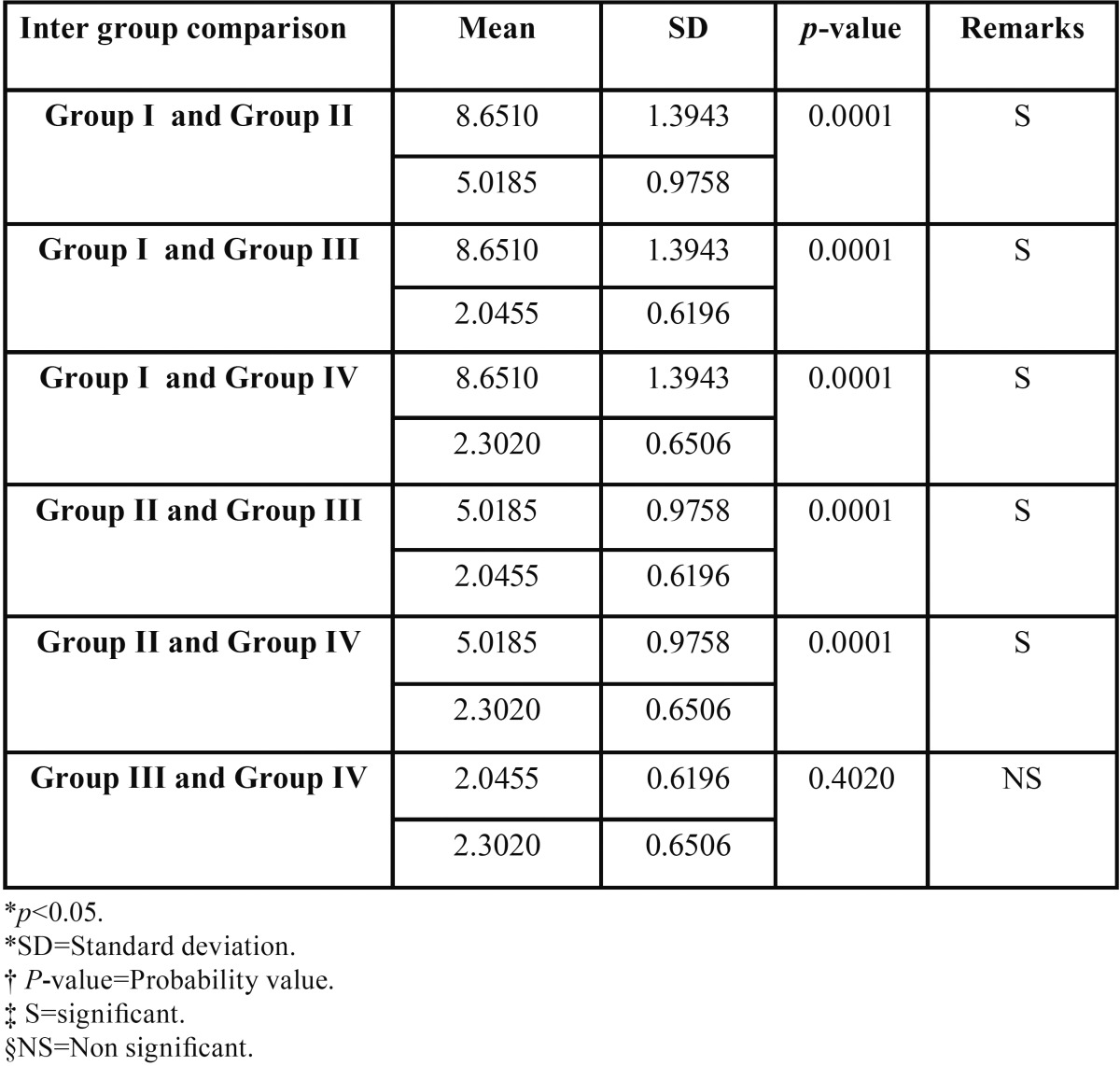
Pair wise comparison of four groups with respect to Shear bond strength (Mpa) by Newman-Keuls multiple post hoc procedure.

## Discussion

Bond failure during orthodontic treatment is relatively frequent and undesirable. As a result, the shear bond strength of new and recycled brackets has been a subject of great interest in orthodontic research (5).

In our study lowest bond strength was obtained for Group III which was flamed and sandblasted (90µm). This is inconsistent with the observations made by Quick AN, Harris AM, Joseph VP who found that sandblasted flamed brackets had no significant effect on shear bond strength of brackets (4). Direct flaming increases the temperature in the range of 600-800°C which leads to disintegration of the metal alloy and weakens its structure which may account for the decreased bond strength.

In our study the highest bond strength was for Group I which consisted of untreated new brackets. This results are in consistent with a study by Samir.E.Bishara who explains that in general, the highest values for shear bond strength were obtained after the initial bonding (5).

In our study Group II sandblasted with 90µm showed higher bond strength among the recycling methods employed. Tavares SW showed that bracket recycling using 90-μm aluminum oxide particle air abrasion enhances bracket bonding to tooth structure by micromechanical retention on base surface and hence increases bond strength compared to 50µm aluminum oxide particle air abrasion (6). In our study sandblasting was done with 90µm aluminium oxide abrasion which produced greater micro-roughness on bracket base surface, increasing considerably the area available for bracket attachment which resulted in highest bond strength among the recycling methods.

In our study there was a significant difference between the shear bond strength of new and recycled brackets. However, Sonis AL 3found no significant difference in shear bond strength between recycled and a new bracket which is contrary to our study result in which the shear bond strength of new brackets is significantly higher than the recycled brackets. This difference may be due to premolar brackets the researcher has used (GAC International, Inc., Central Islip, N.Y.) wheras in our study premolar brackets were manufactured by different company(3M Unitek, Gemini M.B.T, Monorovia, USA).The researcher had carried out sandblasting with brackets bases held approximately 5mm from the tip of microetcher. In our study a distance of 10mm was used to sandblast the brackets and this may account for the variability in the results.

The magnitude of bond strength obtained in the control group (Group I) was more as compared to the bond strength values repor-ted by Quick AN (7.78±1.33MPa and less as reported by Heravi F (12.00±2.47 MPa) in their studies. In the reported study by Quick AN, lower incisor brackets of 0.018” (Mini Diamond Twin, Ormco Corp, California, USA) were used with a mean base area of 8.18mm2 whereas in our study the mean base area is 9.8mm2 and premolar brackets of 0.022” (3M Unitek, Gemini M.B.T, Monorovia, USA) were used in our study. The study did not mention the cross speed at which testing was carried out reporting inconsistent and less shear bond strengths compared to our study.

Heravi F (7) in his study carried out testing at crosshead speed of 5mm/min reporting initial shear bond strength values higher (12.00±2.47 MPa) compared to our present study (8.651±1.3943MPa) where testing was done at a crosshead speed of 0.5mm/min.

Heat itself is a crucial factor in the recycling process because of its negative influence on bracket microstructure. Most orthodontic brackets are made of austenitic stainless steel, which forms chrome-carbide compounds that precipitate at temperatures between 600°C and 800°C. This process leads to disintegration of the metal alloy, weakens its structure and is thus more vulnerable to masticatory damage. In addition to chromium loss via carbide precipitation, corrosion strength also decreases. In our study direct flaming was done using blue flame at temperatures between 600°C and 800°C which could have attributed to decreased bond strength of direct flaming and sandblasted group (Group III) and direct flaming, sandblasted and acid treated group (Group IV).

Sandblasting process is not completely effective in removing the tarnish caused by the flame. Debonded brackets recycled by Bunsen flame are unaesthetic, so the brackets can be treated further to make them more esthetic without compromising the bond strength. We decided to use an acid bath solution to make brackets more esthetic using method suggested by Dawjee S, Gheevarghese O (8) who suggested a simple, quick, and inexpensive way to clean a bracket after the adhesive has been burned off. They submerged the bracket for five to 15 seconds in a solution of 32% hydrochloric acid and 55% nitric acid, mixed in a 1:4 ratio. This process rapidly removes any tarnish, dissolves any adhesive residue, and has a disinfectant effect. A bracket that was recycled with a flame and acid bath solution looks more like a new bracket than one that has been recycled using a flame and microetcher, and therefore would be more esthetically pleasing for the patient. In our study, direct flaming was employed as a part of recycling in Groups III and IV after which the brackets became unaesthetic. We assumed that brackets would become aesthetic along with intention of dissolving residual adhesive, removing tarnish and having a disinfectant effect without affecting the bond strength. However the acid treated brackets in our study became esthetic but the bond strength was significantly reduced.

The shear bond strengths of Group III and Group IV were statistically insignificant. Direct flaming was used for both the groups except that in Group IV brackets were additionally treated with acid bath solution. The acid bath treatment just made the brackets more esthetically acceptable without adding to bond strength. The shear bond strength values of Group III and Group IV were 2.045±0.6196 MPa and 2.3020±0.6506 MPa respectively.

The optimal bond strength required for orthodontic clinical use is as yet unknown (9). Reynolds in 1975 suggested that for an adhesive system to have acceptable clinical performance, *in vitro* bond strength of 5.9-7.8MPa is required (10). Although strong bond that adhesive can offer is desirable in orthodontic practice, bond strength values higher then 9.7Mpa can lead to enamel fractures (11). This study shows that the shear bond strength of new brackets is 8.6510±1.3943MPa which is higher than the optimal bond strength range. In the experimental recycling groups, the sandblasted group (Group II) had the highest bond strength of 5.0185±0.9758MPa which is close to the optimal bond strength value. Group III (2.0455±0.6196 MPa) and Group IV (2.3020±0.6506MPa ) showed too low values to be recommended as preferred chair side recycling methods.

The nature of the forces directed onto orthodontic brackets in the mouth is likely to be a combination of shear, tensile and torsion. The bond strength of bracket -adhesive - enamel system in orthodontic bonding varies and depends on factors such as the type of adhesive, bracket base design, Storage media, enamel morphology, appliance force systems and the clinician’s technique. The universal testing machine used *in vitro* studies is capable of producing only pure debonding forces (shear, tensile or torsion) not the combination of them and other conditions is not possible to simulate. In addition, the rate of loading for the universal testing machine is constant, whereas the rate of loading for *in vivo* debonding is not standardized or constant (12). These are a few among the many factors, which may contribute to the variability and difference of opinion among researchers regarding the clinically acceptable bond strength.

The shear bond strength of sandblasted group (Group II) is close to the available optimal bond strength value in literature. Moreover, recycling techniques used in Group III and Group IV show too low values and cannot be recommended as an effective recycling method. Hence in light of the results presented in our study it can be said that shear bond strength of new brackets is higher than recycled brackets though some inconsistent results have been reported by some researchers (2,13-15). Brackets sandblasted with 90µm aluminium oxide particle air-abrasion was efficient and technically simple, and might provide cost reduction for orthodontists and patients. Sandblasted brackets treated along with flame and acid bath provided no added benefit. In fact, the adjunctive treatment reduced the bracket bond strength.

The nature of the forces directed onto orthodontic brackets in the mouth is likely to be a combination of shear, tensile and torsion. However, in our study just shear forces were evaluated. The rate of loading for *in vivo* debonding is not constant as oral cavity is in a constant dynamic state whereas the rate of loading for the universal testing machine is constant.

To date, however, the clinical bonding performance of the recycled brackets has not been investigated. A prospective, longitudinal *in vivo* clinical study is needed to determine whether recycled brackets can provide clinically acceptable bond strength compared with new brackets.

Conclusions

1. Shear bond strength of new brackets is significantly higher than the recycled brackets.

2. Brackets sandblasted with 90µm aluminium oxide particle air-abrasion showed significantly higher shear bond strength com-pared to direct flaming/sandblasting and direct flaming/sandblasting/acid bath solution.

3. Sandblasting with 90µm aluminium oxide particle air-abrasion is the simplest, most efficient and hence, the preferred method of recycling debonded brackets.
